# Outcomes of Primary Total Hip Arthroplasty Using Custom Femoral Stems in Patients With Secondary Hip Osteoarthritis: A Systematic Review

**DOI:** 10.1016/j.artd.2024.101504

**Published:** 2024-10-12

**Authors:** Kevin Ilo, Prith Hallikeri, Habillan Naathan, Bernard Van Duren, Mark Higgins, Iain McNamara, Toby Smith

**Affiliations:** a Elective Orthopaedics, Nottingham University Hospital, Nottingham, UK; b Norfolk and Norwich University Hospital, Norwich, UK; c Faculty of Medicine and Health Sciences, University of East Anglia, Norwich, UK; d Warwick Medical School, University of Warwick, Coventry, UK

**Keywords:** Custom femoral stems, Secondary hip osteoarthritis

## Abstract

**Background:**

This systematic review aims to evaluate the effectiveness and safety of custom femoral stems in primary total hip arthroplasty (THA) for patients with secondary osteoarthritis with abnormal hip anatomy.

**Methods:**

Following Preferred Reporting Items for Systematic Reviews and Meta-Analyses (PRISMA) guidelines, databases were systematically searched for studies published on primary THA utilizing custom femoral stems. Inclusion criteria were studies on patients with secondary osteoarthritis receiving custom stems, with outcomes including implant survival, revision rates, and functional scores. Data were extracted from eligible studies, with a focus on overall and cause-specific revision rates.

**Results:**

A total of 689 studies were screened, 13 met the inclusion criteria, encompassing 806 patients and 951 custom THA procedures. The collective follow-up period averaged 11.6 years, with a mean age of 44.6 years. The mean reoperation and revision rates were 6.9% (95% confidence interval [CI]: 3.24-10.13) and 8.25% (95% CI: 4.02-12.47), respectively. The mean intraoperative fracture rate was 3.23% (95% CI: 1.35-5.11), and the mean postoperative leg length discrepancy was 4.25 mm (95% CI: 1.57-6.93). The mean improvement of postoperative Harris Hip Score was 40.32 (range 30-56).

**Conclusions:**

Custom femoral stems in primary THA demonstrate promising results in terms of implant survival and functional outcomes for patients with complex hip anatomy due to secondary osteoarthritis. These findings support the consideration of custom implants as a viable option for this patient demographic, although further research is warranted for long-term outcomes and direct comparisons with standard prostheses.

## Introduction

Total hip arthroplasty (THA) is increasingly being utilized to treat younger, more active patients who have developed secondary hip osteoarthritis due to congenital or acquired conditions [1,2]. This poses new challenges in surgical practice and implant design, especially when addressing patients with complex hip anatomy [3]. Cemented femoral stems have been the preferred solution for addressing femoral abnormalities due to their versatility and flexibility during surgery to recreate a patient's normal hip biomechanics [4,5]. However, there are concerns regarding the durability of cemented fixation in younger and more active patients [6,7]. Furthermore, recent studies have highlighted an increased risk of periprosthetic fractures with certain designs of cemented femoral stems [[8], [9], [10]]. As a result, cementless and biological fixation is desirable [11]. Custom cementless femoral stems have the potential to address such issues, especially for patients with femoral deformities. Achieving primary stability is crucial for THA success, but it can be challenging with standard cementless femoral stems, especially in the presence of anatomical irregularities as the proximal femur has a wide range of anatomical variations [12]. These variations make it difficult to achieve an optimal fit-and-fill of the metaphysis with commercially available prostheses, despite the availability of various anatomical designs and sizes [13].

**Figure 1:**
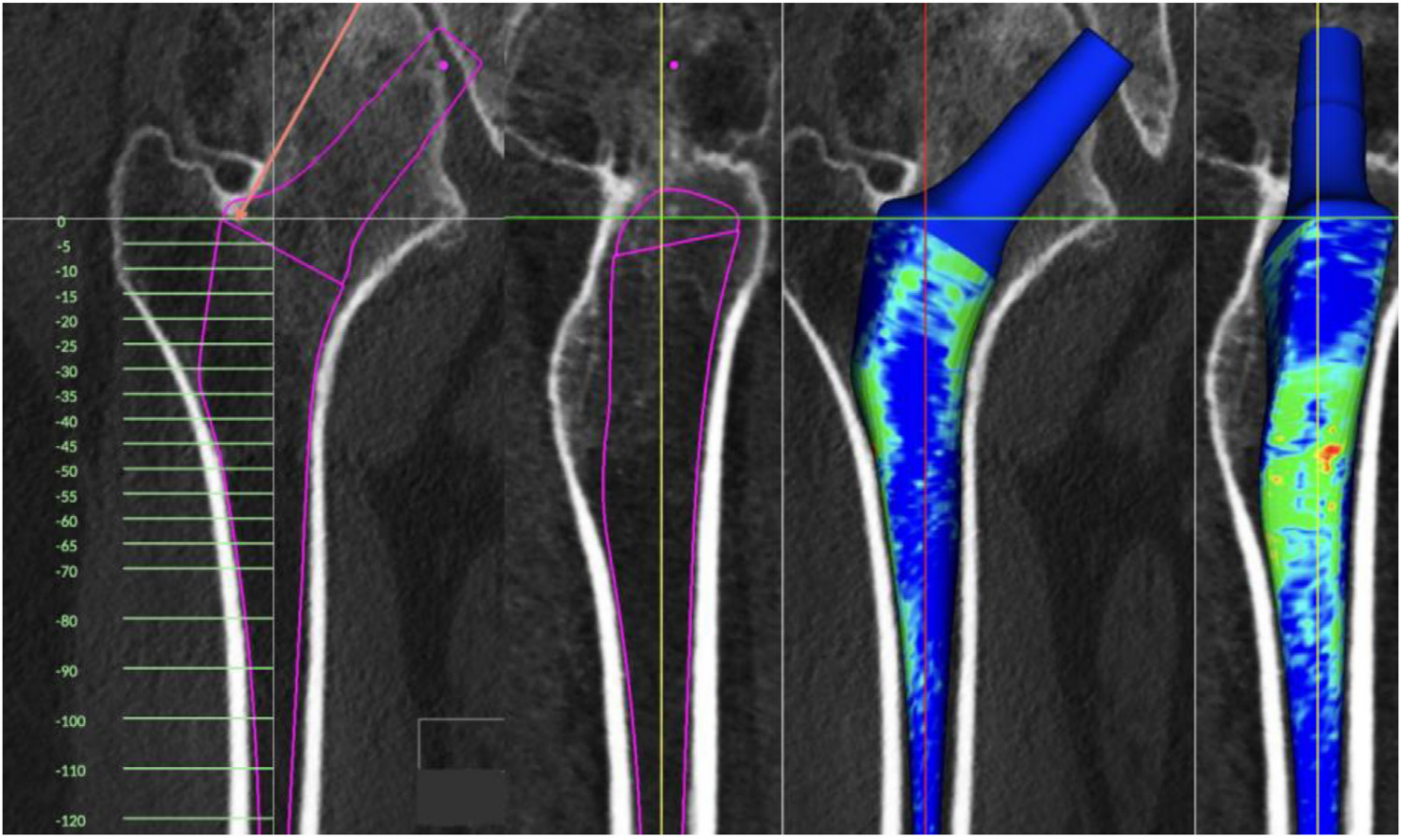
Design of a custom femoral stem utilizing computer tomography imaging for abnormal proximal femur anatomy.

Custom femoral stems, designed and tailored through advanced preoperative three-dimensional (3-D) imaging techniques, are a promising solution (Fig. 1). They have shown considerable utility in treating a range of conditions, including primary osteoarthritis, osteoarthritis secondary to abnormal anatomy, and revision surgery [[14], [15], [16], [17]]. By tailoring the design to the individual's specific anatomy, custom stems ensure a more precise fit, recreating normal hip mechanics and stability, in theory improving their overall outcome [[18], [19], [20], [21]]. For patients with femoral deformity and a long-life expectancy, custom cementless femoral stems represent an encouraging alternative to standard femoral stems. This approach addresses the unique challenges posed by the patient's anatomy and age, offering a solution that aligns more closely with their physiological requirements.

Despite the potential of custom femoral stems, there is a lack of comprehensive clinical outcomes data for custom femoral stems in primary THA, especially in patients with abnormal hip anatomy. This review aims to address this need by examining the clinical outcomes associated with the use of custom femoral stems in secondary hip osteoarthritis, focusing on patients with abnormal hip anatomy and deformity and exploring their benefits and challenges in modern orthopedic practice.

## Material and methods

This systematic review adheres to the guidelines outlined in the Preferred Reporting Items for Systematic Reviews and Meta-Analyses (PRISMA) [22] and has been registered with PROSPERO (Registration: CRD42023488321).

### Search strategy

The search strategy involved an electronic literature search conducted on November 1, 2023, encompassing Medline, Embase, Cochrane, and CINAHL databases. The search terms, including variations of "custom," "stem," and "total hip arthroplasty," were crafted to identify relevant studies (full search strategy in supplemental material). In addition to database searches, reference lists of selected articles and trial registries were scrutinized to identify further relevant studies.

### Eligibility criteria

Inclusion criteria comprised studies reporting clinical outcomes of custom femoral stems designed from preoperative 3-D imaging in secondary hip osteoarthritis. Studies where the majority of the population group (>50%) was diagnosed with primary osteoarthritis were excluded. The exclusion criteria also encompassed non-English studies, those published before 2000, revision THA studies, custom femoral stems not made with 3-D imaging, cemented stems, narrative reviews, expert opinions, and case reports.

The titles and abstracts of all references from the search results were screened for inclusion by 2 independent reviewers (KI, PH). These authors then reviewed the full text of the studies, and disagreements between the 2 reviewers were resolved through review and consensus with a third reviewer (HN).

### Data extraction

Three reviewers (KI, PH, HN) independently reviewed each study and extracted relevant review data. This included year of publication, population characteristics, indication for surgery, type of stem, surgical approach, follow-up duration, type of acetabulum component, and patient demographics. Outcomes including revision rates, reoperation rates, postoperative leg length discrepancies, survival rates of the femoral stem and both components, and preoperative and postoperative patient-reported outcomes were also collected.

### Outcomes

The primary outcome was reoperation events. Secondary outcomes included revision and survival, intraoperative complications, postoperative complications, leg-length discrepancy, patient-reported outcome measures, and health resource use/cost-effectiveness analysis data.

### Critical appraisal

The quality of each study was evaluated using the Joanna Briggs Institute Checklist, an appraisal tool for case series which is an approved method to assess the methodological quality of these studies [23]. This checklist consists of 10 questions, and a point was scored for each, giving a maximum of 10 points. Assessments were performed by one reviewer (KI) and independently verified by 2 other reviewers (HN and PH).

### Data synthesis

Outcomes from the studies were recorded. Arithmetic and weighted means were calculated. Data extraction tables were reviewed for study heterogeneity. Where there was substantial heterogeneity in study design, population characteristics, and surgical procedure, a narrative analysis was performed. Continuous data were assessed using a mean difference and presented with 95% confidence intervals (CIs). Dichotomous data were assessed with relative risk and presented with 95% CI. All data were analyzed using Prism 10 (Prism 10, GraphPad Software, San Diego, CA).

## Results

### Search results

**Figure 2:**
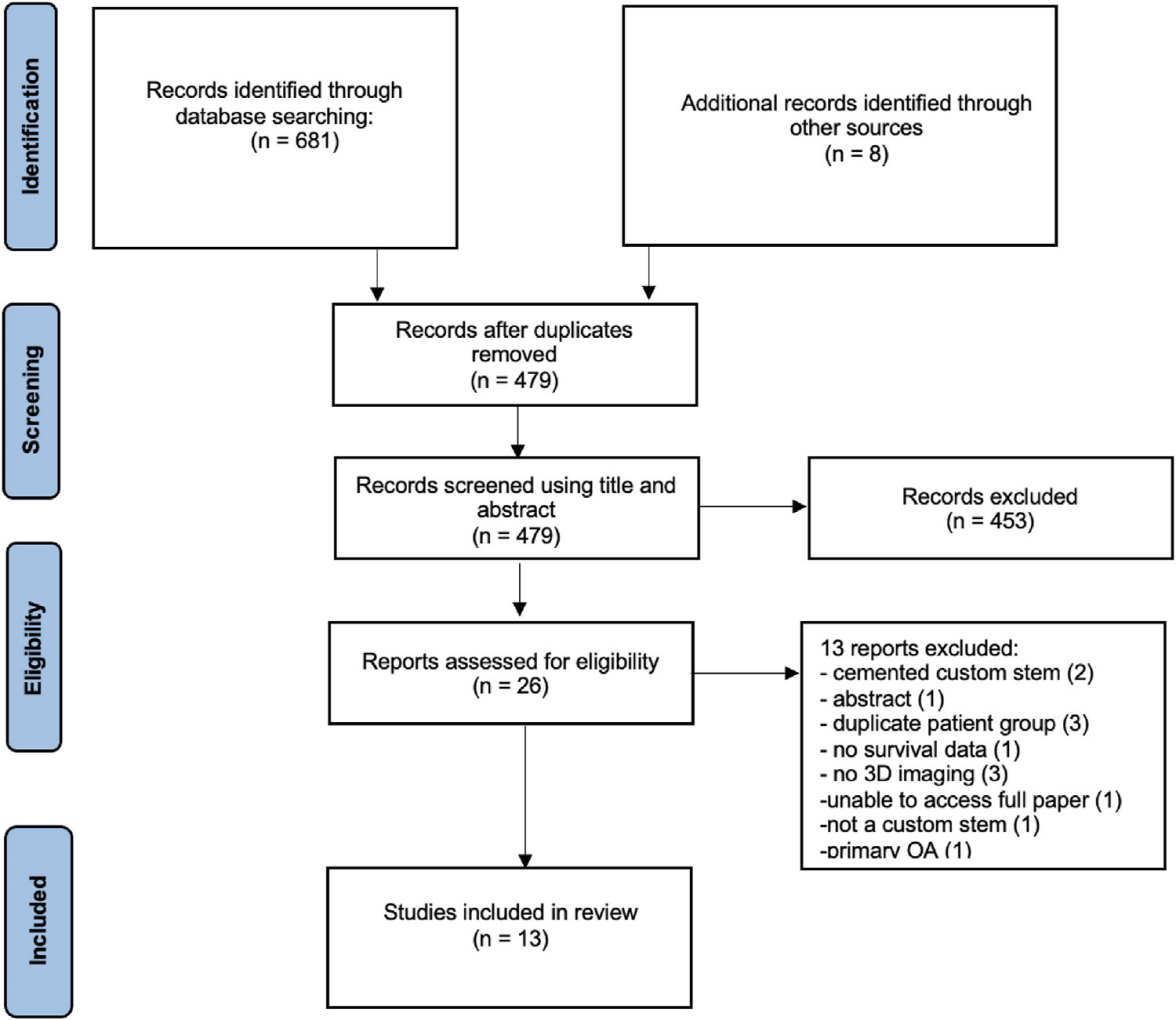
PRISMA flow diagram.

A total of 689 studies were identified, and of these, 202 were duplicates (Fig. 2). A further 41 studies were removed as they were carried out prior to the year 2000 and not in English. The remaining 438 studies were screened using title and abstract. This resulted in the inclusion of 51 studies for full-text screening. Out of these, 13 studies met the inclusion criteria in the systematic review. All 13 studies reported on the clinical outcomes of primary THA using custom stems designed from 3-D imaging in patients with secondary hip osteoarthritis. One study (Jacquet et al.) reported on 2 series of patients [24]. All included studies were case series [17,[24], [25], [26], [27], [28], [29], [30], [31], [32], [33], [34], [35]].

### Study characteristics

The included studies exhibited a mean follow-up duration of 11.6 years (95% CI: 9.48-13.74). The collective patient pool across the studies comprised 806 individuals and 951 custom femoral THAs (Table 1). The mean number of custom femoral stems included in each study was 67.9 (95% CI: 36.1-99.8). The mean age of patients who received a custom femoral stem was 44.6 years (95% CI: 38.4-50.9) with a mean BMI (4 studies included the mean BMI) of 25.4 kg/m^2^ (95% CI: 23.7-27.0). The indications for THA in each study are summarized in Table 1. There were no studies that compared patients with secondary hip osteoarthritis to patients with primary hip osteoarthritis. There were also no studies that compared custom femoral stems to off-the-shelf stems.

**Table 1:** Characteristics of included studies.

Study	No. of patients	No. of hips	Indication	Approach	Mean age (range)	Male no. (%)	Mean BMI (range)
Jacquet et al. 2020 [24]	212	233	Primary OA 17.6%, Secondary OA 49.4% AVN 33% DDH 37.8%, Post-traumatic 11.6%	Anterolateral	39.6 (20-50)	106 (50%)	25 (16-48)
	21	26	DDH (Crowe 3 and 4)	Anterolateral	45 (17-73)	13 (61.9%)	27.2 (16-52)
Flecher et al. 2018 [25]	23	23	Hip fusion	Watson Jones	49 (28-69)	13 (56.6%)	25 (19-33)
Pakos et al. 2015 [35]	67	86	DDH	Posterolateral	Median 48	7 (10.50%)	Median 26.81
Akbar et al. 2009 [26]	61	72	Dysplasia 34.7% Hip dislocation 11.1% AVN 11.1% OA 2.8% Post-traumatic 16.7% Perthes 11.1% RA 9.7% SUFE 2.8%	Anterolateral	35 (22-40)	33 (54%)	26 (18-41)
Flecher et al. 2007 [27]	79	97	Congenital hip dislocation Crowe 1 = 38.1% Crowe 2 = 28.9% Crowe 3 = 13.4% Crowe 4 = 19.6%	Watson Jones	48 (17-72)	5 (6.3%)	Not stated
Al-Khateeb et al. 2014 [28]	14	15	Perthes	Anterolateral or posterior	32.8 (23-55)	6 (42.9%)	Not stated
Koulouvaris et al. 2008 [29]	38	48	Congenital dislocation of hip	Posterolateral	47 (22-69)	Not stated	Not stated
Benum et al. 2010 [33]	83	83	Primary OA 19% Dysplasia 57% Perthes 12% RA 6 Post-traumatic 1% AVN 2% Other 2%	Direct lateral	46 (20-60)	36 (43.4%)	Not stated
Sakai et al. 2006 [17]	77	99	Congenital hip dysplasia Crowe 1 = 47.5% Crowe 2 = 41.4% Crowe 3 = 11.1&	Posterolateral	54 (40-73)	7 (9.1%)	23.6 (17.3-30.6)
Sewell et al. 2011 [30]	25	40	Skeletal dysplasia	Anterolateral or Posterior,	37.5 (18-61)	15 (60%)	Not stated
McCullough et al. 2006 [31]	25	42	Inflammatory polyarthropathy	Not stated	21 (11-35)	7 (28%)	Not stated
Kawate et al. 2009 [34]	53	55	Dysplastic hips	Posterolateral	60 (40-73)	5 (9.4%)	Not stated
Masuda et al. 2016 [32]	28	32	Dysplastic hips with previous osteotomy	Posterolateral	62 (29-77)	2 (7.1%)	Not stated

AVN, avascular necrosis; DDH, developmental dysplasia of hip; OA, osteoarthritis; RA, rheumatoid arthritis; SUFE, slipped upper femoral epiphysis.

### Design of custom stems

Of the 13 included studies of custom femoral stems, there were 6 different manufacturers, and in one study, the manufacturer was not stated (Supplementary Material). All custom stems were designed from computer tomography imaging. They were all uncemented, and 11 studies specified a coating with hydroxyapatite; however, not always stating whether fully or partially. In addition, 10 studies mentioned the material of the femoral stem (titanium alloy), while others did not provide specific material details. The lengths of the custom femoral stems were stated in 4 studies.

### Survival rates, revisions, and reoperations

Eleven studies (N = 780) presented reoperation rates (Table 2). At a mean follow-up of 11.6 years, the overall mean reoperation rate was 6.9% (95% CI: 3.24-10.13). The range of reoperation rates in the studies was 0%-16%. The overall weighted mean reoperation rate was 5.6%.

**Table 2:** Rates and reasons for revision and reoperations in included studies.

Study	Follow-up period in years (range)	Revisions (%)	Reason for revision	Reoperations (%)	Reason for reoperation
Jacquet et al. 2020 [24]	20 (14-27)	23 (9.9%)	Cup -7 for AL -6 for PE wear Both implants -3 for AL -7 for infection	12 (5.2%)	4 infections 3 symptomatic HO 1 PP femur fracture 1 liner dislocation 1 painful trochanteric wire 1 GT fracture non-union 1 dislocation
	16 (10-22)	6 (23.1%)	Cup -2 for dislocation -1 for AL Stem -for PP fracture -2 for AL	1 (3.8%)	1 PP femur fracture
Flecher et al. 2018 [25]	15 (9-22)	1 (4.35%)	Stem -1 for AL	2 (8.7%)	1 infection 1 head fracture
Pakos et al. 2015 [35]	10.6	8 (9.30%)	Cup -3 for AL -1 for PE liner wear Stem -2 for AL Both implants -2 for infection	3 (3.5%)	2 dislocations 1 HO
Akbar et al. 2009 [26]	14 (10-16)	3 (4.17%)	Cup -3 for AL	Not stated	Not stated
Flecher et al. 2007 [27]	10.25 (83-182)	6 (6.2%)	Cup -2 for AL -2 for dislocation Stem -1 for stem fracture Both implants -1 for infection	1 (1.0%)	1 dislocation
Al-Khateeb et al. 2014 [28]	10.1 (5-15)	3 (21%)	Cup -3 for AL	2 (13.3%)	1 symptomatic HO 1 infection
Koulouvaris et al. 2008 [29]	6 (4-8)	3 (6.25)	Cup -1 for mechanical failure Both implants -2 for infection	2 (4.2%)	1 dislocation 1 symptomatic HO
Benum et al. 2010 [33]	10	2 (2.41%)	Stem -2 for PP fracture	7 (8.4%)	1 PP femur fracture 4 PE wear 2 pain
Sakai et al. 2006 [17]	9.25	1 (1.01%)	Stem -1 for AL	Not stated	-Not stated
Sewell et al. 2011 [30]	10.1 (4.3-18.2)	4 (10%)	Cup -2 for AL Stem -1 for infection Both implants -1 for AL	4 (16%)	1 dislocation 2 intraoperative fracture 1 infection
McCullough et al. 2006 [31]	11.2 (8-13)	4 (9.5%)	Cup -2 for AL Stem -2 for AL	6 (14.3%)	1 stem subsidence 4 exchange PE line 1 PP fracture
Kawate et al. 2009 [34]	7 (5-11)	0	0	1 (1.8%)	1 dislocation
Masuda et al. 2016 [32]	13 (10-19)	-	-	3 (9.38%)	3 dislocation

AL, aseptic loosening; GT, greater trochanter; HO, heterotopic ossification; PE, polyethylene; PP, periprosthetic.

Eleven studies (N = 780) presented their revision rates (Table 2). At a mean follow-up of 11.5 years, the overall mean revision rate for custom femoral THA prostheses was 8.25% (95% CI: 4.02-12.47). The range of revision rates in the studies was 0%-23.10%. The overall weighted mean revision rate was 7.0%.

Kaplan–Meier survival was reported in 11 studies (Table 3). Stem survival with aseptic loosening as an endpoint was reported in 11 studies (N = 869). Eight studies (N = 488) [[26], [27], [28], [29],[32], [33], [34], [35]] reported this as 100% with a follow-up ranging from 6 to 14 years. Three studies (n = 381) [17,24,25] reported survival of 87.5%-99%, with a follow-up ranging from 9.3 to 20 years.

**Table 3:** Kaplan–Meir survival data for included studies.

Study	Stem revision for aseptic loosening		Revision of any component for any reason	
	Follow-up years	KM survival (95% CI)	Follow-up years	KM survival (95% CI)
Jacquet et al. 2020 [24]	20	96.8% (95.1-98.5)	20	77.7% (72.4-84)
	15	87.5% (76.5-99.1)	15	72.60%
Flecher et al. 2018 [25]	15	95.6% (92.4-98.8)	Not stated	Not stated
Pakos et al. 2015 [35]	10	100%	10	95.4%%
Akbar et al. 2009 [26]	14	100%	14	86% (64-95)
Flecher et al. 2007 [27]	13	100%	13	89.5% (89.2-89.8)
Al-Khateeb et al. 2014 [28]	10.1	100%	10.1	79%
Koulouvaris et al. 2008 [29]	6	100%	Not stated	Not stated
Benum et al. 2010 [33]	10	100%	Not stated	Not stated
Sakai et al. 2006 [17]	9.3	99% (0.97-1)	9.3	99% (0.97-1)
Sewell et al. 2011 [30]	Not stated	Not stated	Not stated	Not stated
McCullough et al. 2006 [31]	Not stated	Not stated	12	71.4%
Kawate et al. 2009 [34]	7	100%	Not stated	Not stated
Masuda et al. 2016 [32]	13	100%	Not stated	Not stated

CI, confidence interval; KM, Kaplan–Meir.

### Intraoperative fractures and leg-length discrepancy

Intraoperative fracture rates were reported in 11 studies (N = 595) with a mean rate of 3.23% (95% CI: 1.35-5.11). The overall weighted mean was 3.19%. All cases of intraoperative fractures were treated with cabling, except one which required no intervention.

Leg-length discrepancies postoperatively were reported in 5 studies with a mean discrepancy of 4.25 mm (95% CI: 1.57-6.93). The overall weighted mean was 3.08 mm.

### Postoperative patient-reported outcomes

**Figure 3:**
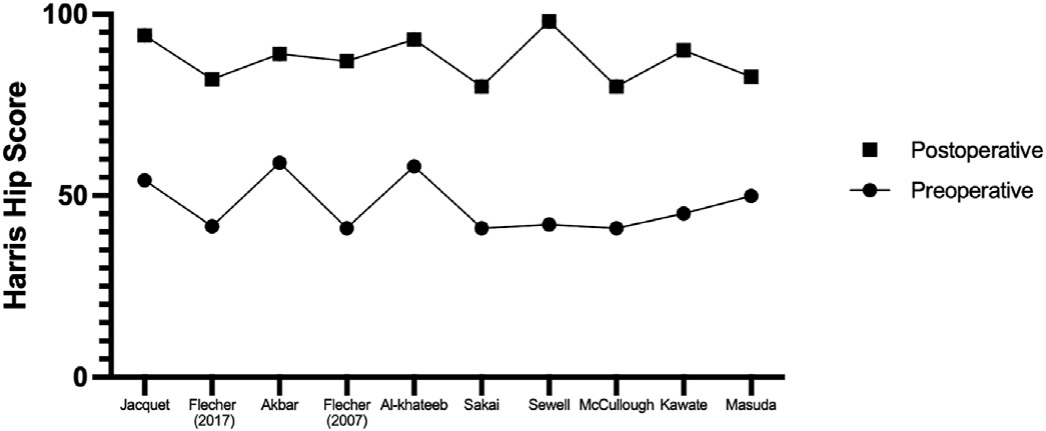
Graph illustrating mean preoperative and postoperative Harris Hip Scores.

All studies included patient-reported outcomes. Ten studies reported their outcomes using Harris Hip Score (HHS; Figure 3) [36], 4 studies presented Merle D’Aubigne scores [37], and one study presented Hospital for Special Surgery system scores [38]. There were insufficient data to permit meta-analysis with studies not reporting inter-quartile range or standard deviation values for specific timepoints. The mean preoperative HHS was 47.26 (range 41-59), and postoperative HHS was 87.58 (range 80-98). The mean improvement in HHS was 40.32 (30-56). The mean preoperative Merle D’Aubigne score was 9 (range 7.6-10), and postoperatively the mean score was 16.63 (range 15.9-17). The one study presenting the hospital for special surgery system score showed an improvement from a median 14 preoperatively to a median 30 postoperatively.

### Quality of evidence

The Joanna Briggs Institute score, reflecting the quality of evidence, indicated a mean score of 6.08 points out of a maximum of 10, with a range of 3 to 9 points (Table 4). Consistently reported strengths in the literature included clearly reported follow-up results of cases (N = 12; 92.3% studies), clear criteria for inclusion in the case series (N = 11; 84.6% studies), and clear reporting of the study participants’ demographics (N = 12; 92.3% studies). Repeated limitations in the evidence included insufficient methods used for identification of the condition for all participants included (N = 11; 84.6% studies) and the condition was not measured in a standard and reliable way for all participants included (N = 9; 69.2% studies)

**Table 4:** Illustrating Joanna Briggs Institute (JBI) Checklist Score and study type.

Study	JBI checklist score
Jacquet et al. 2020 [24]	6 (non-comparative, retrospective)
Flecher et al. 2018 [25]	4 (non-comparative, retrospective)
Flecher et al. 2018 [25]	7 (non-comparative, retrospective)
Pakos et al. 2015 [35]	8 (non-comparative, prospective)
Akbar et al. 2009 [26]	8 (non-comparative)
Flecher et al. 2007 [27]	7 (non-comparative, retrospective)
Al-Khateeb et al. 2014 [28]	9 (non-comparative, prospective)
Koulouvaris et al. 2008 [29]	0 (non-comparative, prospective)
Benum et al. 2010 [33]	8 (non-comparative, prospective)
Sakai et al. 2006 [17]	7 (non-comparative, retrospective)
Sewell et al. 2011 [30]	5 (non-comparative)
McCullough et al. 2006 [31]	7 (non-comparative)
Kawate et al. 2009 [34]	3 (non-comparative, retrospective)

## Discussion

Custom femoral stems offer tailored solutions for deformed proximal femurs, optimizing fixation for unique anatomical challenges. However, their use has limitations, requiring a balanced consideration of benefits against potential challenges in clinical practice. The findings of this systematic review indicate the impressive performance of custom femoral stems in complex patient groups, where achieving durable fixation in abnormal proximal femoral bone is a concern [39,40]. These custom stems demonstrate excellent survival rates against aseptic loosening, with figures ranging from 87.5% to 100% over follow-up periods of 6-20 years. Multiple studies have emphasized that custom femoral stems excel in achieving enhanced metaphyseal fit and fill, a critical factor in boosting both rotational and axial stability [18,41]. The integration of computer-aided design and manufacturing technologies in crafting these stems has been instrumental in achieving this [42,43]. This approach not only preserves bone mass but also optimizes load distribution across the hip joint, characteristics vital for femoral stems, particularly in complex clinical scenarios [42,44]. While the overall survival rate for all components (considering any cause) is somewhat lower, it remains promising in a challenging patient demographic. Notably, most revisions were related to acetabular issues such as loosening and wear, underscoring known challenges with acetabular fixation and durability in these patients [45,46]. The relatively fewer revisions pertaining solely to the femoral component are reassuring.

Custom femoral stems are designed to achieve optimal fit and fill in the metaphyseal region. This is particularly significant in patients with atypical proximal femoral anatomy, who may also present with abnormal bone quality. Such scenarios inherently raise the possibility of intraoperative challenges, including the risk of fractures and potential discrepancies in limb length, should the custom femoral stem not fit as intended [25,47]. Encouragingly, the incidence of intraoperative fractures in custom stems has been reported to be low, even falling below the reported rate of up to 5% for cementless stems in primary THA [[48], [49], [50]]. This is a noteworthy achievement, considering the complexity of cases involving custom stems. Furthermore, the rates of postoperative leg-length discrepancy with custom stems have also been low. When contrasted with the average discrepancies reported in the literature, which range from 3 to 17 mm, the precision achieved with custom stems is commendable [51]. This suggests that with meticulous surgical planning and technique, the risks typically associated with custom stem implantation, such as intraoperative fractures and leg-length discrepancies, can be effectively mitigated while adequately replicating center of rotation of the femoral head, thereby avoiding impingement and reproducing the original foot progression angle [52,53]. These findings underscore the importance of careful preoperative assessment and planning in ensuring successful outcomes with custom femoral stems in THA.

Custom femoral stem manufacturing has evolved over the past 3 decades, shifting from intraoperative silicone mold crafting to preoperative design using radiographs and 3-D imaging. Manufacturers differ in their approach; some modify off-the-shelf models, while others use detailed imaging for a precise anatomical fit. These stems vary in dimensions, shapes, and materials, reflecting diverse manufacturing practices and necessitating treating each stem as a unique, patient-specific implant. This variability challenges standard classification and comparison, as noted in a previous systematic review [54]. Custom stems, designed based on individual patient anatomy and surgeon preferences, offer unique surgical solutions but face challenges like higher costs and extensive preoperative planning [55,56]. Advancements in technologies such as computer-aided design and manufacturing and 3-D printing are revolutionizing the manufacturing of custom stems, by making the process more efficient and cost-effective. Although the initial cost of these advanced manufacturing techniques might be higher, this could be offset by reduced risk of revision. These techniques offer enhanced precision and customization, which enable surgeons to provide more tailored and patient-specific solutions, particularly in complex cases where standard implants may not be adequate. As we continue to embrace these innovations, the future of hip replacement surgery looks to offer more personalized treatment options in order to significantly improve patient outcomes and satisfaction.

This review highlights that existing research lacks direct comparative studies between custom and standard femoral stems in secondary hip osteoarthritis, limiting understanding of custom designs' benefits in complex scenarios. The decision to utilize a custom femoral stem requires deep knowledge of patient anatomy, standard implant limitations, and custom design benefits and challenges. Expertise in preoperative planning and intraoperative techniques is crucial to reduce complications. Current comparative studies show no significant differences, indicating a need for more robust comparative research involving larger cohorts [18,20,21,[57], [58], [59]]. Future studies should include long-term follow-ups to assess custom stems' performance, durability, and effectiveness in mimicking natural biomechanics.

### Limitations

Our review is not without limitations. The potential for publication bias, the heterogeneity of the included studies, and the variability in methodologies, follow-up periods, and patient demographics across studies may affect the generalizability of our conclusions. Furthermore, there is variability in the design and manufacturing of custom femoral stems that cannot be controlled and may result in a difference of outcomes. Data for each included study were not available, so a meta-analysis of the postoperative patient-reported outcomes could not be performed.

## Conclusions

In conclusion, custom femoral stems in primary THA for secondary hip osteoarthritis offer a potentially accurate and reliable solution that can significantly improve patient outcomes. However, their use requires careful consideration of the individual patient’s anatomy, surgical expertise, and the challenges associated with a custom implant design and manufacturing. Future research should aim to directly compare results and cost-effectiveness of custom and standard femoral stems and provide more robust evidence to guide clinical practice. As orthopedic surgery continues to evolve, the quest for optimal solutions in complex primary THA will undoubtedly fuel ongoing research and innovation.

## Conflicts of interest

The authors declare there are no conflicts of interest.

For full disclosure statements refer to https://doi.org/10.1016/j.artd.2024.101504.

## CRediT authorship contribution statement

**Kevin Ilo:** Writing – review & editing, Writing – original draft, Formal analysis, Conceptualization. **Prith Hallikeri:** Writing – original draft, Data curation. **Habillan Naathan:** Data curation. **Bernard Van Duren:** Writing – review & editing, Writing – original draft, Formal analysis. **Mark Higgins:** Writing – review & editing. **Iain McNamara:** Writing – review & editing. **Toby Smith:** Writing – review & editing, Writing – original draft, Formal analysis, Conceptualization.

## Figures and Tables

**Supplementary Table 1:** Details of custom femoral stems included in studies.

Study	Stem manufacturer	Description
Jacquet et al. 2020 [24]	Symbios Ti-Alloy, HA coated	No further details
Flecher et al (2017)	Symbios Ti-Alloy, HA coated	Fitting intramedullary proximal femoral anatomy and accommodating neck offset to the new center of the joint for patient according to the 3-D CT-based preoperative planning
Flecher et al. 2018 [25]	Symbios Ti-Alloy, HA coated	Thick layer coating of porous hydroxyapatite at the proximal part. The HA layer was air plasma sprayed and had a thickness of 75 ± 25 μm. All femoral stems were designed to restore the prosthetic neck anteversion to a normal of 15°. The median femoral neck angle was 130° (IQR: 126°–133°) and the median neck length was 48 mm (IQR: 41.75 mm–56.00 mm). The offset of the prosthesis was calculated according to the opposite hip. If abnormal opposite hip, a 4 cm offset for small patients with narrow pelvis and a 4.5 cm offset for heavy, obese patients.
Pakos et al. 2015 [35]	OS orthopaedic services, GmbH, CT3D-A femoral stem, Ti-alloy, proximal HA-coated	Filling and fitting in the proximal metaphysis. Distal diaphyseal fixation was avoided by reducing the diameter of the stem. The length of the stem ranged from 140 to 160 mm. The macro-structure with a medial bridge and arched structure effectively strengthens both the axial and the rotational stability. A coating layer of hydroxyapatite (HA) (thickness, 80–150 μm) was applied to the proximal two-thirds of the implant.
Akbar et al. 2009 [26]	- Titanium, HA coated	Fitting the intramedullary proximal femoral anatomy and accommodating the offset of the femoral neck to obtain the correct hip center was then inserted the femoral component was designed to produce proximal loading on the femur and was tapered distally. The mean prosthetic neck-shaft angle was 131.8° (102°-143°)
Flecher et al. 2007 [27]	Centre for Biomechanical Engineering, UCL Ti-alloy, HA coated	It had a collar proximally and a lateral flare. The intertrochanteric portion had numerous macro-grooves measuring 1.5 mm in depth and 3 mm in width to increase the surface area for osseointegration. The distal end had longitudinal cutting flutes with a polished finish at the tip to optimize insertion. The implant surface was plasma-sprayed and coated with highly crystallized HA.
Al-Khateeb et al. 2014 [28]	-	The custom prosthesis has a custom grit-blasted broach, which is undersized by 2 mm and is used for impaction of the cancellous bone of the femoral canal.
Koulouvaris et al. 2008 [29]	Unique; Scandinavia customized prosthesis, Trondheim, Norway Proximal HA coated	Designed with a neck that gives a femoral neck anteversion of 10° after insertion unless surgeon decides otherwise. Stems were grit-blasted, and the proximal part of the stem was covered with HA only (CAM Implants, Leiden, The Netherlands) and sterilized by gamma technique (Gamma-Master BV, The Netherlands)
Benum et al. 2010 [33]	Cremascoli, Milano, Italy TI-Alloy, sandblasted	Maximum proximal canal filling. The mean stem length was 121 mm (range, 103–135 mm). Curved anatomic shape with semicylinder-shaped surface grooves with a radius of 0.5 mm were added to the implant in a 5 mm grid to enhance mechanical locking onto the bone, femoral components were sandblasted with mesh sand (Al_2_O_3_, 106–250 um) under 4 bar pressure for a few minutes at room temperature to provide a 4.95 ± 1 μm surface finish.
Sakai et al. 2006 [17]	Centre for Biomechanical Engineering, UCL (assoc. Stanmore implants) Ti-Alloy and proximally HA coated	Collared, lateral flare, intertrochanteric portion had numerous macrogrooves measuring 1.5 mm in depth and 3 mm in width to increase the surface area for osseointegration. The distal end had longitudinal cutting flutes with a polished finish at the tip to optimize insertion. The implant surface was plasma-sprayed and coated with highly crystallized hydroxyapatite.
Sewell et al. 2011 [30]	Centre for Biomedical Engineering, UCL proximally HA coated	All had HA proximal third, high-crystallization HA was used with a thickness of 75 μm to 100 μm. in the first 14 hips, straight-stem femoral components were used, while the subsequent 28 implants had the addition of a proximal lateral flare.
McCullough et al. 2006 [31]	Expert stem version 1 Japan Medical Material, Ti-Alloy and proximally HA coated	To obtain the proximal fixation, the distal part was gradually tapered. The stems did not have collars and the proximal one third was coated using 400μm thick porous coating covered with 20μm HA coating. The average stem length was 10cm (range, 8.7-11.4cm) and average diameter was 8.8 mm (range 6.9-11.8mm)
Kawate et al. 2009 [34]	Expert stem version 1 Japan Medical Material, Ti-Alloy and proximally HA coated	collarless and the proximal third was coated with a 400μm thick porous coating covered with 20μm HA coating. The centre third was coated using sand blasted coating. Average stem length 10.5cm (range 8.9 – 18.8)

Ti, titanium; HA, hydroxyapatite; 3-D, three dimensional; CT, computer tomography.

## Supplementary Material

### Electronic database search strategy

(custom OR custom∗ OR “patient specific”)

AND (stem OR stems)

AND (“total hip arthroplasty” OR“total hip replacement” OR“total hip implants” OR THA OR THR)

## Appendix A Supplementary data

Conflict of Interest Statement for Duren

Conflict of Interest Statement for Naathan

Conflict of Interest Statement for McNamara

Conflict of Interest Statement for Ilo

Conflict of Interest Statement for Higgins

Conflict of Interest Statement for Hallikeri

Conflict of Interest Statement for Smith
